# PET/MR for predicting extranodal extension of head and neck cancer

**DOI:** 10.1007/s00234-025-03635-9

**Published:** 2025-05-21

**Authors:** Vanessa Sanchez, Daniele A. Pizzuto, Alexander Maurer, Urs J. Muehlematter, Bert-Ram Sah, Lars Husmann, Stephan Skawran, Caecilia E. Mader, Gregoire B. Morand, Simon A. Mueller, Christian Meerwein, Niels J. Rupp, Sandra Freiberger, Martin Lanzer, Michael Messerli, Martin W. Huellner

**Affiliations:** 1 Department of Nuclear Medicine, University Hospital Zurich, University of Zurich, Zurich, Switzerland; 2 Nuclear Medicine Unit, GSTeP Radiopharmacy, Fondazone Policlinico Universitario A. Gemelli IRCCS, Rome, Italy; 3 Department of Diagnostic, Interventional, and Pediatric Radiology, University Hospital of Bern, Inselspital, University of Bern, Bern, Switzerland; 4 Department of Nuclear Medicine, Hospital Rechts der Isar, Technical University of Munich, Munich, Germany; 5 Department of Otorhinolaryngology, Head and Neck Surgery, University Hospital Zurich, University of Zurich, Zurich, Switzerland; 6 Department of Pathology and Molecular Pathology, University Hospital Zurich, University of Zurich, Zurich, Switzerland; 7 Department of Cranio-Maxillo-Facial and Oral Surgery, University Hospital Zurich, University of Zurich, Zurich, Switzerland

**Keywords:** PET/MR, Head and neck cancer, Hybrid imaging, Extranodal extension, 2- [18F]-fluorodeoxy-D-glucose

## Abstract

**Purpose:**

To analyze the diagnostic accuracy of multiparametric FDG-PET/MR in identifying pathologic extranodal extension (pENE) of lymph node metastases (LNM) in head and neck squamous cell carcinoma (HNSCC) patients.

**Methods and materials:**

Retrospective analysis of 57 HNSCC patients who underwent preoperative FDG-PET/MR imaging. PET parameters of LNM SUVmax and MTV, lymph node size as well as MR parameters flare sign, shaggy margin sign and vanishing border sign were analyzed. Histopathological assessment of neck dissection specimens served as standard of reference.

**Results:**

A logistic regression model consisting of lymph node size (*p* = 0.029), shaggy margin sign (*p* = 0.031) and MTV (*p* = 0.035) proved that all three parameters significantly contributed to the prediction of pENE (χ²(3) = 54.23, *p* < 0.001). A second model without the reader-dependent parameter shaggy margin sign yielded similar results (χ²(2) = 45.36, *p* < 0.001), with every increase in lymph node size (*p* = 0.006) by 1 mm increasing the likelihood of pENE by a factor of 1.41 (95%-CI[1.11, 1.81]), and every increase in MTV (*p* = 0.023) by 1 cm3 increasing the likelihood of pENE by a factor of 1.64 (95%-CI[1.07, 2.50]). This model yielded an accuracy of 94.7% (95%-CI [85.4, 98.9]) for predicting pENE, with a specificity of 97.3% (95%-CI [85.8, 99.9]) and a sensitivity of 90.0% (95%-CI [68.3, 98.8]). Internal validation using a test dataset confirmed high accuracy of this model.

**Conclusion:**

PET/MR-based multivariate binomial logistic regression models consisting of MTV, lymph node size and/or shaggy lymph node margins predict pENE with high accuracy.

## Introduction

Extranodal extension on histopathology (pENE) of lymph node metastases (LNM) represents an important prognostic indicator in head and neck squamous cell carcinoma (HNSCC). Pathologically, it is characterized by the infiltration of metastatic tumor cells through the lymph node’s capsule with or without invasion of adjacent structures [1–3]. Approximately 60% of nodal-positive HNSCC patients exhibit pENE 4. Oropharyngeal HNSCC can be categorized into two histopathologically distinct subtypes: p16-positive and p16-negative tumors. P16 is a protein utilized as a surrogate marker for high-risk human papillomavirus (HPV)-mediated carcinogenesis, which significantly impacts patient prognosis. HPV-positive HNSCC typically exhibit a more favorable prognosis [5–8], with their outcomes appearing to be less influenced by the presence of ENE compared to HPV-negative HNSCC patients [9].

The presence of LNM reduces the 5-year survival rate by 50%. Additionally, the presence of pENE further decreases survival rates and is correlated with an increased likelihood of locoregional recurrence and distant metastases 2, 10. Consequently, pENE has been incorporated into the Union for international cancer control (UICC) TNM system following the 2016 revision and into the American joint committee on cancer (AJCC) system in 2018. In both classifications, pENE corresponds to the nodal stage N3b in HPV-negative tumors (with the exception of single nodes smaller than 3 cm, which corresponds to stage pN2a), while pENE today is not considered in the N staging of HPV-positive tumors [1, 4, 11, 12, 13].

The management of HNSCC involves a multidisciplinary approach. The presence of pENE typically warrants adjuvant radiochemotherapy [14, 15]. Therefore, accurate staging, especially precise nodal staging, is crucial early in the diagnostic process. This is particularly significant given that upfront surgery is not always the preferred approach, and the oncologic outcome may ultimately not vary significantly [16–21]. Neck dissection specimens serve as the standard of reference for determining pENE [22]. These enable both macroscopic and microscopic examination, providing precise information on extracapsular growth 20. In instances where there is insufficient material for histopathology, extranodal extension can be evaluated using subjective and reader-dependent imaging criteria on computed tomography (CT) or magnetic resonance (MR). Radiological ENE (rENE) on MR can be approximated by irregular margins (shaggy margin sign), increased signal intensity surrounding the lymph node capsule (flare sign), or inhomogeneity of the adjacent adipose tissue (vanishing border sign) [1, 23, 24].

2-[18F]-fluorodeoxy-D-glucose (FDG) positron emission tomography (PET) with either CT or MR as anatomical imaging component has been proven valuable, as hybrid PET imaging is highly sensitive for detecting LNM and distant metastases [25, 26]. Meta-analyses comparing hybrid imaging PET/CT and PET/MR mostly demonstrate comparable results, with some studies suggesting superior performance of PET/MR in T staging and N staging for certain HNSCC locations [27–29]. However, specific studies on PET/MR imaging for specific HNSCC subtypes and its role in (radio)therapy planning are still limited [30, 31]. One significant advantage of hybrid PET imaging is the provision of quantitative, user-independent and reproducible data. Recent studies, such as those by Morand et al. [32] or Werner et al. [33], demonstrate the potential prediction of ENE using nodal metabolic PET parameters, such as the maximum standardized uptake value (SUV_max_) and metabolic tumor volume (MTV), which has also been demonstrated for other tumor entities like breast and lung cancer [34, 35]. Hybrid imaging also facilitates the elimination of discrepancies arising from temporal changes, which are a constraint of sequential modalities [22, 36]. Therefore, the objective of our study was to analyze the diagnostic accuracy of FDG-PET/MR in identifying pENE of lymph node metastases in HNSCC patients.

## Materials and methods

### Study design and patients

Approval for this retrospective study was obtained by the local ethics committee and all included patients provided written informed consent to the utilization of their medical data for research purposes. The cohort comprised 57 consecutive newly diagnosed HNSCC patients retrieved from the radiological information system, who underwent preoperative staging with FDG-PET/MR between 01/2015 and 09/2018, followed by neck dissection. The primary tumor location encompassed 4 different sites with a variable number of subsites, and CUP. Characteristics of the cohort are detailed in Table 1. The interval between imaging and surgery with neck dissection varied from 3 to 20 days (median: 9 days). Histopathology of lymph nodes served as standard of reference in all patients. Patient staging in our study adhered to the UICC TNM protocol for head and neck cancer, 8th edition 2017 ^11, 21^.

**Table 1:** Demographics and overview of primary tumor locations. CUP, cancer of unknown primary. * if not specified otherwise

Characteristic	Number* (frequency*)
Age in years (median (range))	66 (39–95)
Female	16 (28.1%)
Male	41 (71.9%)
**Primary tumor location**	
Oropharynx	
Base of tongue	13 (22.81%)
Multiple sites	9 (15.79%)
Palatine tonsil	8 (14.04%)
Soft palate	1 (1.75%)
Vallecula	1 (1.75%)
Oral cavity	
Tongue margin	5 (8.77%)
Alveolar ridge	1 (1.75%)
Hypopharynx	12 (21.05%)
Larynx	
Supraglottis	2 (3.51%)
Glottis	1 (1.75%)
CUP	3 (5.26%)

### PET/MR image analysis

PET/MR imaging was conducted using a Signa 3T PET/MR scanner (GE HealthCare, Waukesha, WI). Imaging followed detailed protocols previously published, comprising a focused scan covering the head and neck, as well as a whole-body scan [37, 38].

The following MR pulse sequences were utilized to assess rENE: T1WI (T1-weighted image, non-enhanced in axial plane), fsT2WI (fat-suppressed T2-weighted image in axial plane) and fat-saturated ceT1WI (Gadolinium contrast-enhanced T1-weighted image in axial, coronal and sagittal plane). The size of the largest lymph node or lymph node conglomerate was determined on ceT1WI (maximum long axis diameter in any plane). The following PET parameters of the largest lymph node or lymph node conglomerate were recorded: SUV_max_ and MTV. SUV_max_ serves as a surrogate parameter for the lymph node’s glucose metabolism representing the maximum FDG uptake and is depicted as the hottest voxel on the PET image. MTV represents the metabolically active volume of a tumor, specifically the sum of voxels exceeding an SUV_max_ threshold, which was defined as 42% in our study, being in line with previous research [2, 4, 19, 22, 30, 33, 34].

### Radiologic extranodal extension on MR images

The assessment of the largest lymph node or lymph node conglomerate was conducted by two readers (D.A.P., M.W.H.) with 14 and 12 years of experience, respectively, in radiology and nuclear medicine. In cases where their findings diverged, a consensus decision was reached. To determine the presence of rENE, readers scrutinized the images for the following features (present / absent): (1) *vanishing border sign* on T1WI (i.e., haziness in the adipose tissue between metastatic lymph node and adjacent tissue, or obliterated adipose tissues spaces between a node and surrounding structures), (2) *flare sign* on fsT2WI (i.e., presence of high-intensity signal in interstitial tissue adjacent to and extending from a lymph node) and (3) *shaggy margin sign* on ceT1WI (i.e., irregular or jagged margin of a lymph node) [1, 23, 39].

The largest lymph node was selected as it provides a straightforward and consistent reference point, facilitating retrospective correlation with pathological reports to ensure accurate matching with cross-sectional imaging data. Clinically, the precise number of lymph nodes exhibiting extranodal extension (ENE) is less significant, as the presence of even a single node with ENE is sufficient to influence the N classification and guide treatment decisions [13, 39, 40].

### Pathologic extranodal extension

Histopathological examination was conducted following established and standardized in-house procedures. Data was extracted from pathological reports. Assessment of pENE was categorized into macroscopic and microscopic levels (threshold of 2 mm), with the latter considered the standard of reference for our study. pENE was identified if the specimen exhibited growth beyond the lymph node capsule into the surrounding adipose tissue or other adjacent tissue. Nodal HPV status was determined either by the presence of p16 expression in immunohistochemical examination and/or via polymerase chain reaction (PCR) HPV analysis of the probes [2, 22, 41].

### Statistical analysis

The distribution of continuous variables was assessed for normality following Gauss’s theorem. Median and interquartile range are reported for all continuous variables (age, T classification, lymph node size, SUVmax, MTV). Given that all continuous variables except age did not follow a normal distribution, the distribution of pENE among samples was compared using Mann-Whitney U-test. Based on the three morphological MR parameters (flare sign, shaggy margin sign, vanishing border sign), two additional variables were created: MR2pos (two or more of the three MR parameters positive), MR3pos (all three MR parameters positive). A binomial logistic regression was conducted to ascertain the impact of age, sex, T classification, p16 status, lymph node size, morphological MR parameters and PET parameters on predicting the likelihood of pENE. Linearity was assessed using the Box-Tidwell procedure [42]. Bonferroni correction was applied to all seventeen terms in the model as per Tabachnik and Fidell [43]. Only variables significant in univariate analysis were entered into the final model. All variables entered into the model were tested for multicollinearity. A p-value of < 0.05 was considered to indicate statistical significance. Statistical analysis used SPSS statistics version 29.0 (IBM, Armonk, NY). Based on these models, heat maps and nomograms were calculated. An internal test dataset was used for validation.

## Results

Of the 57 lymph nodes analyzed, 20 (35.1%) exhibited pENE, while 37 (64.9%) did not. pENE was purely microscopic in 12 (21.1%) nodes, and macroscopic in 8 (14.0%) nodes. 41 of the 57 patients (71.2%) had HPV-negative tumors, and 16 patients (28.8%) had HPV-positive tumors. The median time interval between PET/MR imaging and surgery was 9 days (interquartile range, 5 days). The vanishing border sign was present in 21 (36.8%) of the analyzed lymph nodes, the flare sign was present in 23 lymph nodes (40.4%), and the shaggy margin sign was present in 28 (49.1%) lymph nodes.

### Univariate analysis and logistic regression

**Fig. 1:**
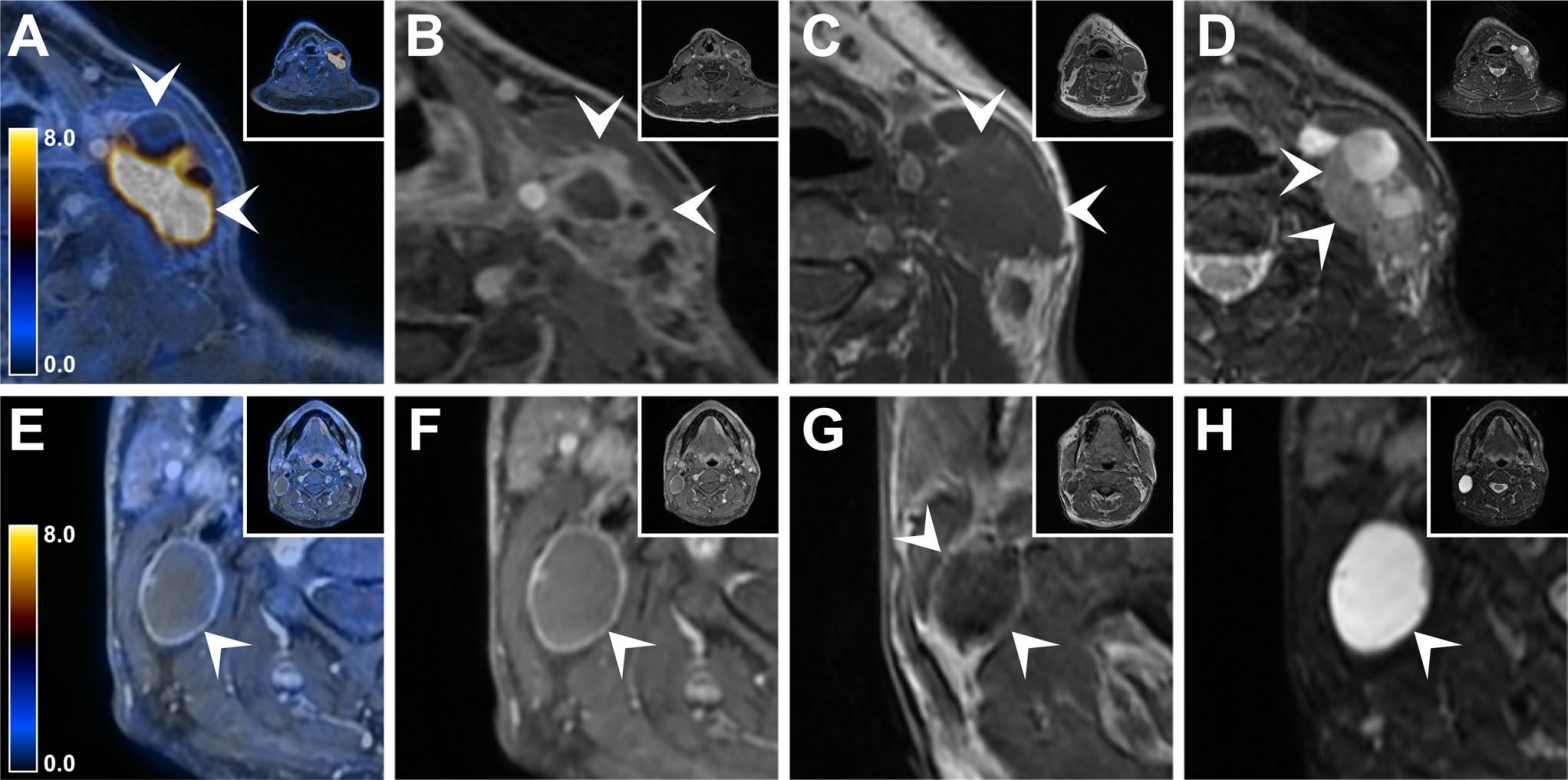
FDG-PET/MR imaging features in patients with and without ENE. Panels **A) - D)** 54-year-old man with hypopharyngeal carcinoma and histopathologically confirmed extranodal extension of lymph node metastases. **(A)** Axial fused FDG-PET/MR shows a left-sided partially cystic / necrotic cervical lymph node metastasis with intense FDG uptake (SUV_max_ 17.7, MTV 7.2 cm^3^, white arrowheads). **(B)** Axial contrast-enhanced, fat-suppressed, T1-weighted MR image illustrates ENE of the lymph node metastasis, infiltrating adjacent structures (shaggy margin sign, white arrowheads). **(C)** On axial T1-weighted MR image, the lymph node metastasis cannot be reliably distinguished from adjacent structures (vanishing board sign, white arrowheads). **(D)** Axial fat-suppressed T2-weighted MR image reveals small hyperintense protrusions along the border of the lymph node metastasis (flare sign, white arrowheads). Panels **(E - H)** 57-year-old man with tonsillar carcinoma and absence of extranodal extension on histopathology. **E)** Axial fused FDG-PET/MR shows a right-sided cervical lymph node metastasis with faint FDG uptake (SUV_max_, 2.2, MTV 3.9 cm^3^, white arrowheads). **(F)** Axial contrast-enhanced, fat-suppressed, T1-weighted MR image demonstrates a clear delineation of the border and the absence of extranodal extension of the lymph node metastasis (white arrowhead). **(G)** On axial T1-weighted MR image, the borders of the lymph node metastasis can be accurately distinguished from neighboring structures (white arrowhead). **(H)** Axial fat-suppressed, T2-weighted MR image illustrates the mainly cystic nature of the lymph node metastasis featuring a smooth border (white arrowhead)

According to the Mann-Whitney U-test, the variables lymph node size, all three morphological MR parameters, the derived parameters MR2pos and MR3pos, as well as the two PET parameters exhibited significant differences (*p* < 0.001) concerning pENE (Table 2; Fig. 1). Consequently, these variables were entered into the binomial logistic regression model for pENE. On the other hand, the variables age, sex, T classification and p16 status did not demonstrate significant differences and were therefore not included in the model.

**Table 2:** **Parameters in univariate analysis.** pENE, pathologic extranodal extension; IQR, interquartile range; MTV, metabolic tumor volume; SD, standard deviation; SUV_max_, maximum standardized uptake value. * if not indicated otherwise

Variables (median (IQR))*	pENE absent	pENE present	*p*-value
Age [years]	67 (12)	60 (17)	0.128
T classification	3 (3)	3 (2)	0.965
Lymph node size [mm]	15 (10)	27 (15)	< 0.001
Vanishing border sign (mean (SD)	0.16 (0.37)	0.75 (0.44)	< 0.001
Flare sign (mean, SD)	0.14 (0.35)	0.90 (0.31)	< 0.001
Shaggy margin sign (mean, SD)	0.24 (0.44)	0.95 (0.22)	< 0.001
SUV_max_	5.82 (5.83)	12.7 (7.03)	< 0.001
MTV [cm^3^]	1.85 (3.25)	7.6 (10.58)	< 0.001

Using an MTV threshold of 6.5 cm^3^ yielded a diagnostic accuracy of 0.807 (95% CI [0.681–0.900]) with a sensitivity of 0.600 (95% CI [0.361–0.809]) and a specificity of 0.919 (95% CI [0.781–0.983]) for the prediction of pENE. An SUV_max_ threshold of 10.3 yielded a diagnostic accuracy of 0.872 (95% CI [0.763–0.949]) with a sensitivity of 0.850 (95% CI [0.621–0.968]) and a specificity of 0.892 (95% CI [0.746–0.970]) for the prediction of pENE.

### Prediction of pathologic ENE – general model

According to the Box-Tidwell [42] procedure, all variables were found to follow a linear relationship. Due to multicollinearity, all variables except lymph node size, MTV, and shaggy margin sign were excluded from the model. The binomial logistic regression model yielded statistical significance (χ²(3) = 54.23, *p* < 0.001), resulting in a large amount of explained variance according to Backhaus et al. [44], as shown by Nagelkerke’s R² = 0.845. Goodness-of-fit was assessed using the Hosmer-Lemeshow test, revealing a satisfactory model fit (χ²(8) = 2.74, *p* > 0.05). The overall percentage of accuracy in classification was 89.5%, with a sensitivity of 85.0% and a specificity of 91.9%. All three variables entered into the regression model significantly contributed to predicting pENE: lymph node size (*p* = 0.029), shaggy margin sign (*p* = 0.031) and MTV (*p* = 0.035).

All model coefficients and odds are presented in Table 3. For every increase in lymph node size by 1 mm, the likelihood of ENE increased by a factor of 1.39 (95%-CI [1.03, 1.86]). Similarly, for every increase in MTV by 1 cm^3^, the likelihood of ENE increased by a factor of 2.07 (95%-CI [1.05, 4.06]).

**Table 3:** General model - overview of model coefficients and odds. B, Β regression coefficient; CI, confidence interval; MTV, metabolic tumor volume; SE, standard error of mean

					95% CI for Odds ratio	
	B	SE	*p*-value	Odds ratio	lower bound	upper bound
Lymph node size	0.326	0.150	0.029	1.386	1.034	1.859
MTV	0.726	0.345	0.035	2.067	1.052	4.062
Shaggy margin	4.101	1.896	0.031	60.371	1.468	2483.424
Constant	-13.368	5.098	0.009	0.000		

### Prediction of pathologic ENE – reader-independent model

Since the shaggy margin sign as macroscopic imaging parameter is subject to reader-dependent interpretation and would be used to predict microscopic ENE (although macroscopic ENE per definition includes also microscopic ENE), a second binomial logistic regression model was constructed, incorporating only the presumably less reader-dependent parameters MTV and lymph node size [45–47].

This second binomial logistic regression model was also demonstrated statistical significance (χ²(2) = 45.36, *p* < 0.001), resulting in a large amount of explained variance according to Backhaus et al. [44], as shown by Nagelkerke’s R² = 0.755. Goodness-of-fit was assessed using the Hosmer-Lemeshow test, revealing a satisfactory model fit (χ²(8) = 3.717, *p* > 0.05). The overall percentage of accuracy in classification was comparable to the previous model (87.7%, with a sensitivity of 80.0% and an identical specificity of 91.9%). Both variables entered into this regression model significantly contributed to predicting pENE: lymph node size (*p* = 0.006), MTV (*p* = 0.023).

The model coefficients and odds are detailed in Table 4. For every increase in lymph node size by 1 mm, the likelihood of ENE increases by a factor of 1.41 (95%-CI [1.11, 1.89]). Similarly, for every increase in MTV by 1 cm^3^, the likelihood of ENE increases by a factor of 1.64 (95%-CI [1.07, 2.50]).

**Table 4:** Reader-independent model - overview of model coefficients and odds. B, Β regression coefficient; CI, confidence interval; MTV, metabolic tumor volume; SE, standard error of mean

					95% CI for Odds ratio	
	B	SE	p-value	Odds ratio	lower bound	upper bound
Lymph node size	0.346	0.126	0.006	1.414	1.105	1.808
MTV	0.492	0.216	0.023	1.635	1.070	2.497
Constant	-9.911	3.307	0.003	0.000		

**Fig. 2:**
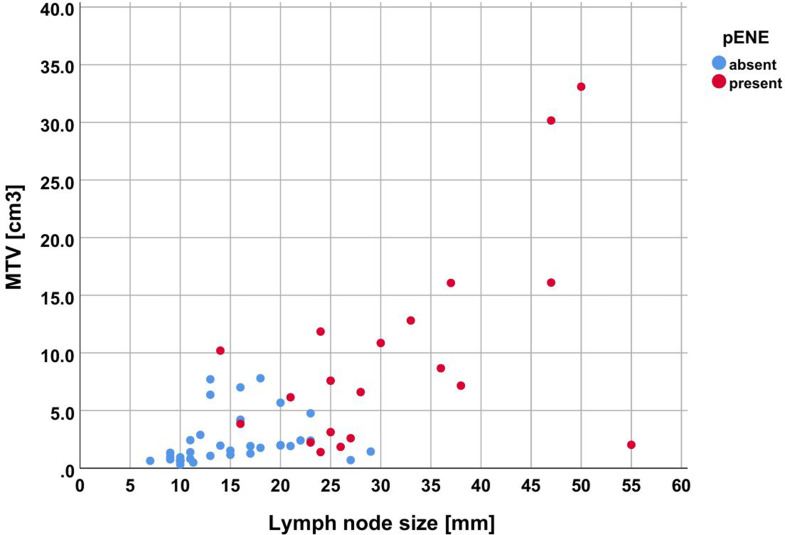
Dot plot representing the lymph node metastases by size and MTV, categorized by presence of pENE

Visual data inspection using contingency tables and cross tabulation revealed that using an MTV threshold of 10.0 cm^3^ in lymph nodes sized up to 20 mm (*n* = 33), an MTV threshold of 5.0 cm^3^ in lymph nodes sized 21–23 mm (*n* = 6), and an MTV threshold of 1.0 cm^3^ in lymph nodes larger than 23 mm (*n* = 18) yields an accuracy of 94.7% (95%-CI [85.4, 98.9]) for predicting pENE, with a specificity of 97.3% (95%-CI [85.8, 99.9]) and a sensitivity of 90.0% (95%-CI [68.3, 98.8]) (Fig. 2).

### Internal validation, heat maps and nomograms for external usage

**Fig. 3:**
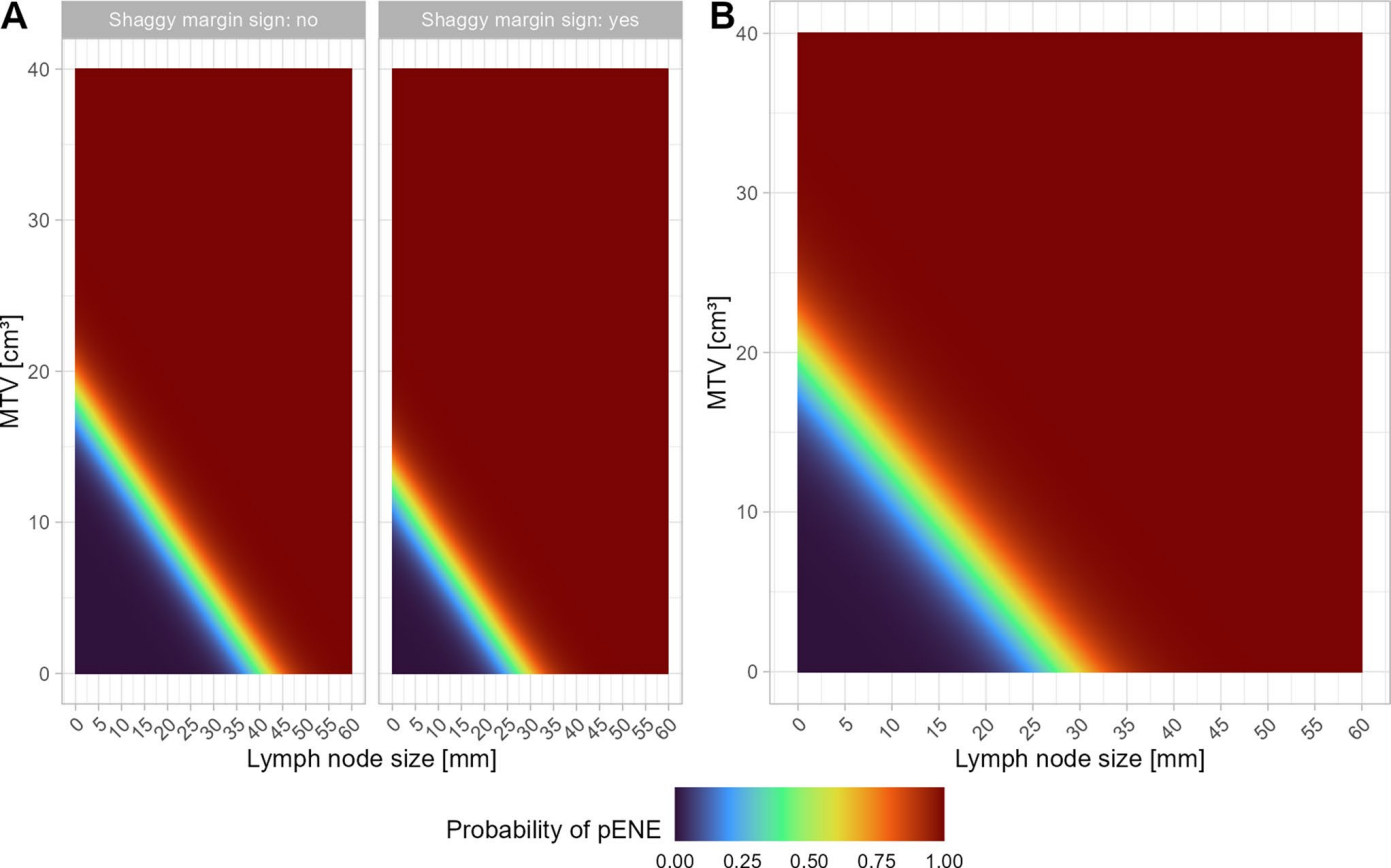
Logistic regression analysis-derived color-coded maps indicating the probability of pENE. **(A)** Heat map derived from the general model with shaggy margin sign, MTV, and lymph node size. **(B)** Heat map derived from the reader-independent model with MTV and lymph node size. MTV values greater than 40 cm³ and lymph node sizes greater than 60 mm are truncated for improved visualization

**Fig. 4:**
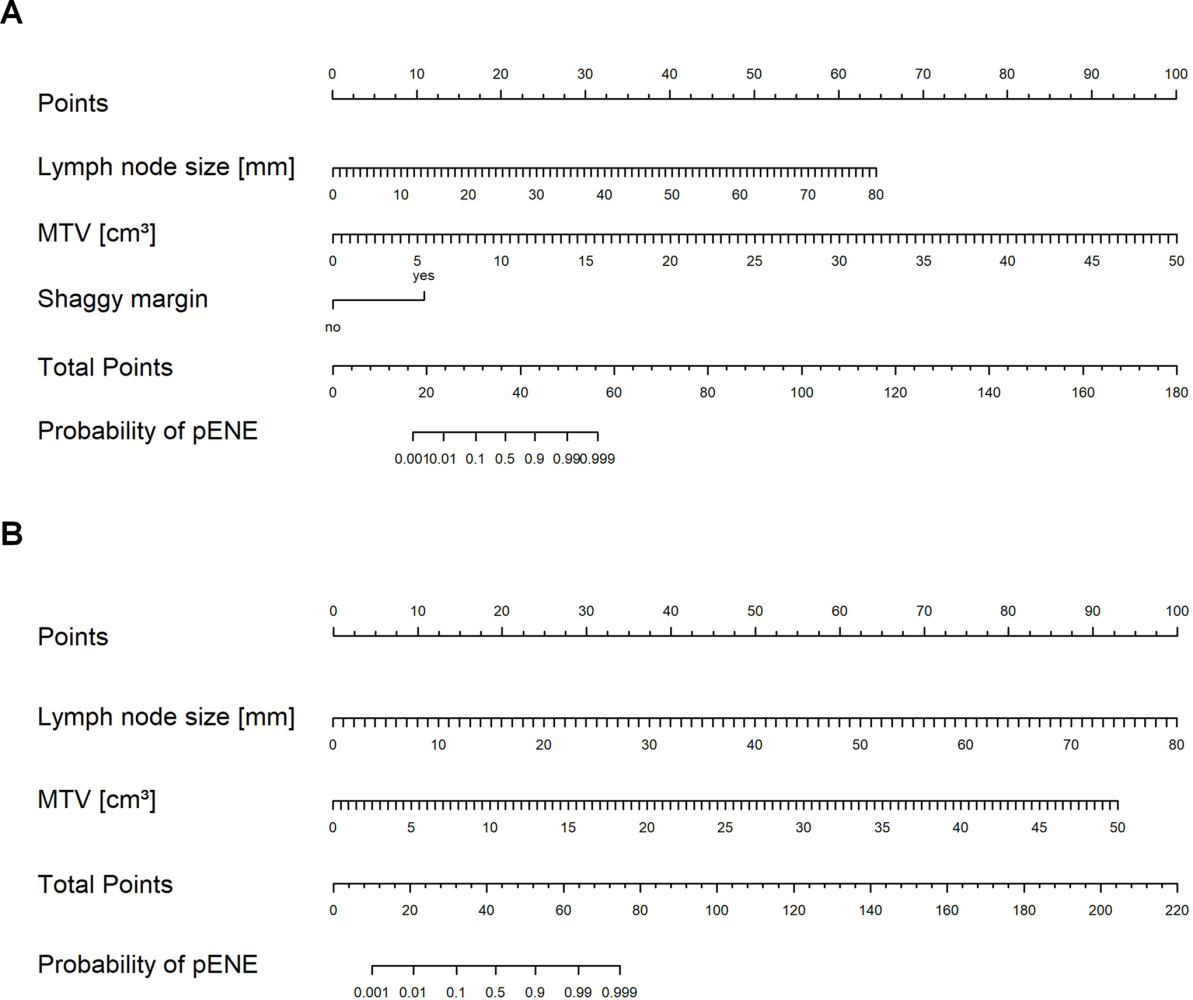
Logistic regression analysis-derived nomograms indicating the probability of pENE. **(A)** Nomogram derived from the general model with shaggy margin sign, MTV, and lymph node size. **(B)** Nomogram derived from the reader-independent model with MTV and lymph node size

Based on the logistic regression analyses, color-coded maps (heat maps) were generated for the general model (Fig. 3A) and the reader-independent model (Fig. 3B). Additionally, nomograms (Fig. 4) were created to serve as resources for external usage. The reader independent model was validated against an internal dataset of 50 different patients who underwent PET/MR imaging between 10/2018 and 04/2022. Using the suggested model yielded an accuracy of 90.0% (95%-CI [78.2, 96.7]) for predicting pENE, with a specificity of 85.0% (95%-CI [62.1, 96.8]) and a sensitivity of 93.3% (95%-CI [77.9, 99.2]) (negative predictive value 89.5% (95%-CI [68.8, 97.0]), positive predictive value 90.3% (95%-CI [76.6, 96.4])). Harrell’s C-index was 0.80.

## Discussion

The aim of our retrospective study was to assess the potential of non-invasive, multiparametric FDG-PET/MR in predicting the presence of histopathological extranodal extension in HNSCC patients with lymph node metastases. We employed binomial logistic regression models to identify parameters significantly associated with the presence of pENE, facilitating early and accurate diagnosis. Our main finding was that a model consisting of MTV and lymph node size predicts pENE with high accuracy, which was validated internally.

A binomial logistic regression model consisting of lymph node size, shaggy margin sign and MTV proved that these three parameters may predict pENE (χ²(3) = 54.23, *p* < 0.001). For every increase in lymph node size by 1 mm, the likelihood of pENE increases by a factor of 1.39 (95%-CI [1.03, 1.86]). For every increase in MTV by 1 cm^3^, the likelihood of pENE increases by a factor of 2.07 (95%-CI [1.05, 4.06]). A second model without the reader-dependent parameter shaggy margin sign yielded similar results (χ²(2) = 45.36, *p* < 0.001), with every increase in lymph node size (*p* = 0.006) by 1 mm increasing the likelihood of pENE by a factor of 1.41 (95%-CI[1.11, 1.81]), and every increase in MTV (*p* = 0.023) by 1 cm^3^ increasing the likelihood of pENE by a factor of 1.64 (95%-CI[1.07, 2.50]). This second model yielded an accuracy of 94.7% (95%-CI [85.4, 98.9]) for predicting pENE.

Our results provide a more comprehensive insight and support findings made in previous studies. For example, Douglas et at. [10] linked a longest lymph node diameter > 30 mm and haziness in adjacent adipose tissue with the presence of ENE. In one of our groups’ previous studies, Nemmour et al. [22] provided support for the significant difference in metabolic parameters observed in PET imaging, showing higher nodal SUV_max_ and MTV in patients with histopathological extranodal extension, in line with our findings [22]. Joo et al. [2] primarily observed significant differences in SUV_max_ for cervical LNM with ENE, while Toya et al. [4] additionally established that an SUV_max_ >3.0 is associated with a high detection rate of ENE in terms of sensitivity and specificity. Kitajima et al. [19] recommended using an SUV_max_ cutoff for evaluating rENE. However, their findings indicated that the sensitivity of hybrid imaging was insufficient to replace histopathological evaluation following neck dissection. While we acknowledge this limitation, having an accurate and largely reader-independent non-invasive test for detecting pENE could aid decision-making in cases where neck dissection is not feasible and thus cannot serve as a standard of reference. Altogether, literature data on specific cut-off values for PET parameters is heterogeneuous and in part contradicting, with a wide range of thresholds. In our study, besides calculating cutoff values for MTV and SUV_max_ for reference purposes, we aimed to identify variables from multiparametric hybrid PET/MR imaging that were significant predictors of pENE. As assessment criteria for rENE have not been universally defined, leading to potential interobserver discrepancies, the results from our second model, which excludes subjective criteria, aimed to mitigate this incongruity.

We acknowledge that the clinical significance of microscopic pENE, which was analyzed in our study, is still under investigation. Macroscopic pENE typically correlates with to clinically evident ENE (defining N3b status in p16-negative tumors), becoming prognostically significant when its extent exceeds approximately 2 mm [48]. Several reliable diagnostic methods, such as MR imaging and clinical palpation, are already available for its detection. However, these tests often discourage the need for neck dissection, limiting access to a more definitive histopathological reference. Therefore, the identification of microscopic pENE via imaging could be an important diagnostic objective, which can be validated through histopathology.

Our models may serve as a decision-making aid for clinicians involved in therapy planning. The proposed MTV-threshold-based approach, which depends on three different lymph node size ranges, is considered easily applicable in clinical routine. In clinical practice, the presence of ENE, for instance, impacts radiotherapy planning, necessitating the consideration of recommendations such as applying a 10 mm nodal clinical target volume margin [4]. In certain cases, complete surgical lymph node dissection may be impractical due to challenging anatomy. Radiological estimation of ENE proves even more valuable in these cases if pathological examinations are not feasible.

Of note, our models do not incorporate the T classification, since this parameter was insignificant at univariate level, despite the fact that tumor staging is based on prognostic implications of depth of invasion, which is known to be associated with an increased risk of nodal metastases and locoregional recurrence [11, 49]. The same was true for the p16 status. Other studies on this topic demonstrated that p16-positive tumors, associated with HPV-positivity, exhibit significantly improved outcomes and distinct histopathological features [6, 8]. This suggests the potential for risk stratification by rENE. The insignificant results of our study concerning this matter may be attributed to our relatively small study cohort (*n* = 57) and the heterogeneity of HNSCC locations including 11 different subtypes, although the majority of tumors originated from well-known p16 predilection sites. The retrospective and single-center design represents another limitation. Selecting the largest node could bias the results for the relationship between ENE and nodal size. However, pathological data from a large cohort of HNSCC does not support this assumption [40]. Limiting our study to include only patients who underwent surgical neck dissection may be regarded as a form of selection bias. Further, our study represents exploratory data analysis proposing a model with no external validation. One limitation of the proposed reader-independent model is the comparably small number of lymph nodes in the medium size range (21–23 mm).

Of note, our reader-independent model using only MTV and lymph node size may also be applicable to PET/CT imaging, which could be investigated in future studies [50]. It remains to be seen if the multiparametric models provided in our study can be generalized to a broader HNSCC population.

In conclusion, PET/MR-based multivariate binomial logistic regression models consisting of MTV, lymph node size and/or shaggy lymph node margins predict pENE with high accuracy. Results of our study may aid pretreatment assessment of HNSCC patients.

## Abbreviations

AJCC: American joint committee on cancer.
Bβ: Regression coefficient.
CI: Confidence interval.
ceT1WI: Contrast-enhanced T1-weighted image.
CT: Computed tomography.
ENE: Extranodal extension.
FDG: 2-[^18^F]-fluorodeoxy-D-glucose.
fsT2WI: Fat-suppressed T2-weighted image.
HNSCC: Head and neck squamous cell carcinoma.
HPV: Human papilloma virus.
IQR: Interquartile range.
LNM: Lymph node metastases.
MR: Magnetic resonance.
MTV: Metabolic tumor volume.
NCCN: National Comprehensive Cancer Network.
PCR: Polymerase chain reaction.
PET: Positron emission tomography.
pENE: Pathologic extranodal extension.
rENE: Radiologic extranodal extension.
SE: Standard error.
SUV_max_: Maximum standardized uptake value.
T1WI: T1-weighted image, non-enhanced.
UICC: Union for International Cancer Control.
